# Psychological Impact of COVID-19 on Ophthalmologists in Iran

**DOI:** 10.18502/jovr.v17i2.10795

**Published:** 2022-04-29

**Authors:** Masomeh Kalantarion, Zhale Rajavi, Hamideh Sabbaghi, Bahareh Kheiri, Mohammad Hasan Shahriari, Farinaz Fatahi Mozafar

**Affiliations:** ^1^Department of Medical Education, Virtual School of Medical Education and Management, Shahid Beheshti University of Medical Sciences, Tehran, Iran; ^2^Ophthalmic Epidemiology Research Center, Research Institute for Ophthalmology and Vision Science, Shahid Beheshti University of Medical Sciences, Tehran, Iran; ^3^Negah Aref Ophthalmic Research Center, Shahid Beheshti University of Medical Sciences, Tehran, Iran; ^4^Department of Ophthalmology, School of Medicine, Shahid Beheshti University of Medical Sciences, Tehran, Iran; ^5^Department of Optometry, School of Rehabilitation, Shahid Beheshti University of Medical Sciences, Tehran, Iran; ^6^Ophthalmic Research Center, Research Institute for Ophthalmology and Vision Science, Shahid Beheshti University of Medical Sciences, Tehran, Iran; ^7^Department of Electrical Engineering, Faculty of Computer Engineering, University of Isfahan, Isfahan, Iran; ^8^Department of Psychology, Kish International Branch, Islamic Azad University, Kish Island, Iran; ^9^These two authors equally contributed to this work.

**Keywords:** Coronavirus Disease, Iran, Ophthalmologists, Psychological Impact

## Abstract

**Purpose:**

To identify the psychological impact of coronavirus disease on ophthalmologists practicing in Iran between August and December 2020.

**Methods:**

In this cross-sectional online survey, a standard Patient Health Questionnaire- 9 (PHQ- 9) was completed by 228 ophthalmologists who were practicing in Iran. The PHQ- 9 questionnaire was revised by adding two additional questions specifically applicable for the assessment of the psychological impact of coronavirus disease on the Iranian ophthalmologists. An organized classification regarding the assessment of different depression severities identified as no (0–4), mild (5–9), moderate (10–14), or severe (15–21) was then considered for data analysis.

**Results:**

The mean age of our participants was 49.0 ± 15.61 years and the majority of them (67.1%) were male. Depression was discovered in 73.68% (*n* = 168) with different severities ranging from mild (*n* = 61, 26.75%), moderate (*n* = 63, 27.63%), and severe (*n* = 44, 19.3%). It was found that participants with depression were older as compared to those without depression (*P* = 0.038). Higher percentages of severe depression were noticed in the high-risk regions contaminated with corona virus as compared to the other low-risk regions (*P* = 0.003). Based on multivariable models, we determined that ophthalmologists who were somewhat concerned about their training/ profession (OR: 0.240; 95% CI: 0.086–0.672; *P* = 0.007) and those with no concerns about their income had lower association with depression (OR: 0.065; 95% CI: 0.005–0.91; *P* = 0.042).

**Conclusion:**

High prevalence of depression was observed among older aged Iranian ophthalmologists living in high-risk contaminated regions who possessed serious concerns with respect to their training/profession and income. It is recommended that the health policymakers of Iran pay more attention to the ophthalmologists who experience the aforementioned factors.

##  INTRODUCTION

Coronaviruses (CoVs) are the known types of viruses affecting both birds and mammals, which developed into a pandemic following the outbreak of the COVID-19.^[1]^ This was first identified in Wuhan, China at the end of 2019 and was later announced as the Public Health Emergency of International Concern by the World Health Organization (WHO) on January 30, 2020.^[2,3,4,5]^ In terms of gender incidence, the disease was more prevalent in males with an estimation of 54.3% and a median age of 56 years in Wuhan, China.^[3]^

Both physical and psychological complications have been reported in patients with COVID-19 virus.^[6,7,8,9]^ In terms of psychological concerns, the medical staff and healthcare workers showed acute stress reactions during the COVID-19 pandemic through emotional, cognitive, physical, and social reactions.^[8]^ Corona-phobia is a public concern which is mostly observed in the majority of healthcare professionals who are in direct contact with quarantined patients in hospitals.^[7]^ Medical workers such as doctors, nurses, and paramedical staff are currently in a stressful working environment due to the lack of suitable protection which makes them at high risk for infection, caring for patients while experiencing negative emotions, working for long hours, and isolation from family and loved ones.^[7]^

Numerous mental disorders attributed to the COVID-19 pandemic have been reported among the healthcare workers. These include increased depression/depressive symptoms, anxiety, psychological distress, and poor sleep quality.^[7]^ These psychological problems such as stress, depressive symptoms, insomnia, denial, anger, fear would result to decreasing the quality of life. These findings were also reported among Chinese medical workers and Italian general practitioners.^[10,11]^

In the current pandemic, due to the ocular manifestations, ophthalmologists have also encountered a high referral rate of patients with COVID-19 virus.^[12]^ They have a potentially high risk of contracting coronavirus infections via transmission of the virus through droplets due to the close proximity needed for examination and the direct contact with patients' lids and ocular surfaces.^[13,14,15]^ These possibilities illustrate that they are working in a highly stressful conditions. Noticeable transient mental health problems including depression, anxiety, and stress were reported among the training and practicing ophthalmologists as well as the ophthalmic surgeons in India.^[14]^ The current pandemic also had an adverse effect on the mental health of the highest record of ophthalmology residents (70.5%) in Saudi Arabia.^[16]^

Due to the prevalence of the COVID-19 epidemic in Iran as well as the increased referral rate of patients to the eye centers, we decided to identify the psychological impact of COVID-19 on ophthalmologists practicing in Iran.

##  METHODS

In this cross-sectional online survey, a standard Patient Health Questionnaire-9 (PHQ-9) was distributed to our target population of ophthalmologists who were practicing throughout Iran. The current study was conducted between August and December 2020.

This study was approved by the Ethics Committee of the Ophthalmic Research Center, Shahid Beheshti University of Medical Sciences (Approval No.: IR.SBMU.ORC.REC.1399.016). A brief explanation regarding our study objectives and the instructions about how to complete the questionnaire were distributed to our target population – all registered ophthalmologists in the Society of Ophthalmology – either by email or via various social media platforms. To ensure adequate and timely feedback from participants they were also sent three reminders. We assured our participants that their information was kept anonymous and confidential.

### Patient Health Questionnaire-9 (PHQ-9)

Data were collected using a standard questionnaire as previously applied in the study by Khanna et al.^[13]^ A Persian version of this questionnaire with the sensitivity of 73.8% and specificity of 76.2% was applied in the present study.^[17]^

In addition to the nine specific questions regarding the assessment of depression, sociodemographic characteristics such as gender, age, marital status, duration of practice, last degree, and type of services offered were also recorded on the questionnaire. The PHQ-9 questionnaire was revised to include three additional questions that were specific to the purpose of this study. Two questions focused on the ophthalmologists' concerns about their training/profession and their income and another question was introduced to clarify the association of the current situation with depression.

Afterward, this was presented to an expert panel including ophthalmologists, psychologists, biostatisticians, and research methodologists to assess its content validity.

The questionnaire was accessible by all participants through the link address: https://docs.google.com/forms/d/e/ 1FAIpQLSfpclV5xi7dZEKrGX_wAwjTh_mdyVoTd5 rXFf3x3M6C7GSFkA/viewform?usp=sf_link.

An organized classification representing different depression severities of no (0–4), mild (5–9), moderate (10–14), or severe (15–21) were then considered for data analysis.^[18,19]^ The prevalence of depression was also reported based on the severity of the COVID-19 contamination in different regions of Iran which were reported by the Iranian National Headquarter for the Control of COVID-19 Epidemic in December 2020.^[20]^

### Statistical Analysis

To analyze the data, we used frequency (%), mean ± SD, median and range. To evaluate the difference between the two groups of the ophthalmologists who had depression and those without it, we used *t*-test, Mann–Whitney, Chi-Square, and Fisher's Exact test. We used binary logistic regression to calculate the OR and effect of each associated factor. The correlation of all considered factors with depression was analyzed based on both univariate and multivariable models, in which all assumptions were considered. A *P*-value < 0.05 was considered as statistically significant. All statistical analyses were performed by SPSS software (IBM Corp. Released 2017. IBM SPSS Statistics for Windows, Version 25.0. Armonk, NY: IBM Corp.).

**Figure 1:**
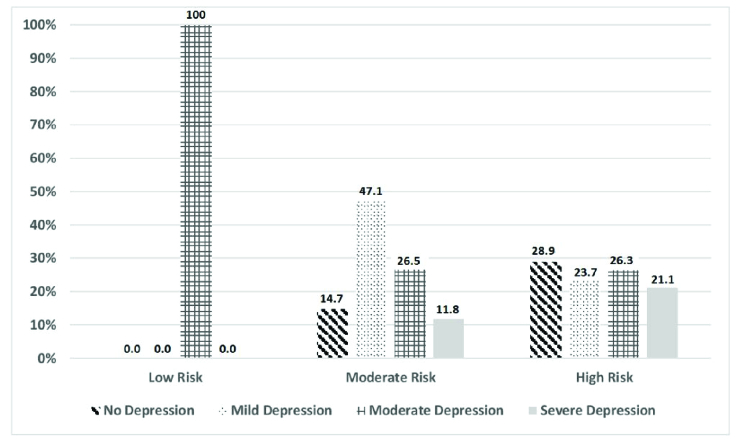
The prevalence of various severities of depression among ophthalmologists working in different regions of Iran in 2020.

**Table 1:** Sociodemographic characteristics of the study population regarding depression


** Factors**		** Level**	**Total**	**Depression**		***P*** **-value**
		**No (** ***n*** ** = 60)**	**Yes (** ***n*** ** = 168)**	
Gender (%)	Male	153 (67.1%)	41 (68.3%)	112 (66.7%)	0.874
		Female	75 (32.9%)	19 (31.7%)	56 (33.3%)	
Age (yr)	Mean ± SD	49 ± 15.61	51.7 ± 10.68	66.25 ± 10.11	0.038
		Median (Range)	40 (31 to 85)	51.5 (31 to 70)	60 (50 to 85)	
Marital status (%)	Married	196 (85.97%)	54 (90.0%)	142 (84.5%)	0.388
		Single	32 (14.03%)	6 (10.0%)	26 (15.5%)	
Duration of practice (yr)		Mean ± SD	17.26 ± 10.86	19.1 ± 10.46	16.6 ± 10.95	0.127
	Median (Range)	18 (0–50)	20 (1–43)	16.5 (0–50)	
Last degree (%)	General Ophthalmologist	98 (42.99%)	20 (33.3%)	78 (46.4%)	0.151
		Fellowship	Cornea & Anterior Segment	46 (20.17%)	10 (16.7%)	36 (21.4%)	
	Vitreoretinal	55 (24.12%)	21 (35.0%)	34 (20.2%)	
	Strabismus	8 (3.50%)	3 (5.0%)	5 (3.0%)	
	Glaucoma	8 (3.50%)	1 (1.7%)	7 (4.2%)	
	Oculoplastic	12 (5.29%)	4 (6.6%)	8 (4.8%)	
	Pathology	1 (0.43%)	1 (1.7%)	0 (0.0%)	
Type of Services (%)		Governmental	115 (50.43%)	31 (51.7%)	84 (50.0%)	0.609
	In training	12 (5.26%)	4 (6.7%)	8 (4.8%)	
	Private	97 (42.56%)	23 (38.3%)	74 (44.0%)	
	NGO	4 (1.75%)	2 (3.3%)	2 (1.2%)	
Concerns about training or profession (%)		Not at all	4 (1.7%)	4 (6.7%)	0 (0.0%)	< 0.001
	Somewhat	64 (28.1%)	29 (48.3%)	35 (20.8%)	
	Considerable	96 (42.1%)	17 (28.3%)	79 (47.0%)	
	Seriously	64 (28.1%)	10 (16.7%)	54 (32.1%)	
Concerns about income (%)		Not at all	5 (2.19%)	4 (6.7%)	1 (0.6%)	0.002
	Somewhat	58 (25.43%)	22 (36.7%)	36 (21.4%)	
	Considerable	88 (38.59%)	20 (33.3%)	68 (40.5%)	
	Seriously	77 (33.77%)	14 (23.3%)	63 (37.5%)	
Probable Associated Factors with Depression (%)	Coronavirus	No	118 (51.75%)	33 (55.0%)	85 (50.6%)	0.652
	Yes	110 (48.24%)	27 (45.0%)	83 (49.4%)	
	Communication Problem	No	94 (41.22%)	20 (33.3%)	74 (44.0%)	0.17
	Yes	134 (58.77%)	40 (66.7%)	94 (56.0%)	
	Previous Psychological Problem	No	205 (89.91%)	58 (96.7%)	147 (87.5%)	0.047
	Yes	23 (10.08%)	2 (3.3%)	21 (12.5%)	
	Financial Problem	No	158 (69.29%)	48 (80.0%)	110 (65.5%)	0.05
	Yes	70 (30.70%)	12 (20.0%)	58 (34.5%)	
	Distress due to losing relatives	No	219 (96.05%)	58 (96.7%)	161 (95.8%)	> 0.999
	Yes	9 (3.94%)	2 (3.3%)	7 (4.2%)	
	Isolation	No	208 (91.22%)	57 (95.0%)	151 (89.9%)	0.296
	Yes	20 (8.77%)	3 (5.0%)	17 (10.1%)	
	Others	No	228 (100%)	60 (100.0%)	168 (100.0%)	–
	Yes	0 (0.0%)	0 (0.0%)	0 (0.0%)	
	
	

**Table 2:** Univariate and multivariable analyses of the probable associated factors with depression among ophthalmologists


**Factors**	** Level**	**Univariate analysis**				**Multivariable analysis**			
	**OR**	**95% CI**		***P*** **-value**	**OR**	**95% CI**		***P*** **-value**
		**Lower**	**Upper**		**Lower**	**Upper**	
Gender	Male	0.927	0.493	1.743	0.814	–	–	–	–
		Female	R	–	–	–	–	–	–	–
Residential Region	High Risk	R	–	–	–	–	–	–	–
	Low Risk	–	–	–	–	–	–	–	–
	Moderate Risk	2.363	0.87	6.42	0.092	0.379	0.014	10.064	0.562
Marital Status	Married	0.607	0.237	1.556	0.298		
	Single	R	–	–	–	–	–	–	–
Last Degree	General Ophthalmologist	R	–	–	–	–	–	–	–
	Cornea & Anterior Segment	0.923	0.392	2.172	0.855	2.653	0.631	11.161	0.183
	Glaucoma	0.427	0.094	1.941	0.271	1.855	0.421	8.174	0.414
	Strabismus	1.795	0.209	15.441	0.594	1.085	0.139	8.458	0.938
	Vitreoretinal	0.415	0.199	0.864	0.019	2.976	0.244	36.367	0.393
	Pathology	_	_	_	_	_	_	_	_
	Oculoplastic	0.513	0.14	1.876	0.313	0.99	0.241	4.068	0.989
Type of Services	Government	2.71	0.366	20.078	0.329	–	–	–
	In training	2	0.201	19.914	0.554	–	–	–	–
	Private	3.217	0.429	24.134	0.256	–	–	–	–
	NGO	R	–	–	–	–	–	–	–
Concerns about training or profession	Not at all	–	–	–	–	–	–	–	–
	Somewhat	0.223	0.097	0.515	< 0.001	0.24	0.086	0.672	0.007
	Considerable	0.861	0.366	2.022	0.73	0.827	0.0.333	2.411	0.827
	Seriously	R				
Concerns about income	Not at all	0.056	0.006	0.536	0.012	0.065	0.005	0.91	0.042
	Somewhat	0.364	0.166	0.797	0.012	1.107	0.441	2.776	0.829
	Considerable	0.756	0.352	1.622	0.472	0.913	0.388	2.471	0.858
	Seriously	R	–	–	–	–	–	–	–
	
	

##  RESULTS

In this cross-sectional online survey, a PHQ-9 questionnaire was completed by 228 ophthalmologists (response rate: 16.9%) with a mean age of 49.0 ± 15.61 years (median, 40.0; range, 31–85) where the majority of the study participants (67.1%) were male.

Depression was assessed in 73.68% (*n* = 168) of the participants with severities ranging from mild (*n* = 61, 26.75%), moderate (*n* = 63, 27.63%), and severe (*n* = 44, 19.3%) among our studied subjects. It was revealed that depressed ophthalmologists were older as compared to those without depression (*P* = 0.038). Additionally, an increased prevalence of depression was identified among ophthalmologists who had either a previous psychological problem (*P* = 0.047) or considerable concerns regarding their training, profession (*P* < 0.001), and their income (*P* = 0.002). Regarding the other sociodemographic factors presented in Table 1, no statistically significant difference was observed between the ophthalmologists with depression versus those without depression.

Figure 1 illustrates the frequency of varied severities of depression in different residential regions of Iran classified based on the level of contamination with the COVID-19 virus. As shown, 42.11% of ophthalmologists practicing in the low- or moderate-risk regions had mild depression, while the higher percentage of the severe depression was found in the high-risk regions as compared to the other regions (*P* = 0.003).

The univariate model shows that ophthalmologists in vitreoretinal fellowship had lower frequency of depression as compared to general ophthalmologists (OR: 0.415; 95% CI: 0.199–0.864, *P* = 0.019). Furthermore, ophthalmologists who were somewhat concerned about their training/profession had a lower association with depression as compared to those having serious concerns (OR: 0.223; 95% CI: 0.097–0.515, *P* < 0.001). This finding was correspondent among ophthalmologists who were somewhat concerned (OR: 0.364; 95% CI: 0.166–0.797, *P* = 0.012) or who had no (OR: 0.056; 95% CI: 0.006–0.536, *P* = 0.012) concerns about their income. Based on multivariable models, we determined that ophthalmologists who were somewhat concerned about their training/profession (OR: 0.240; 95% CI: 0.086–0.672; *P* = 0.007) and those with no concerns about their income possessed lower association with depression (OR: 0.065; 95% CI: 0.005–0.91; *P* = 0.042) [Table 2].

##  DISCUSSION

The current study was purposed to investigate the prevalence of depression among the registered ophthalmologists who were practicing in Iran. Depression was found in 73.68% (*n* = 168) of our studied subjects with severities ranging from mild (26.75%), moderate (27.63%), and severe (19.3%). The study by Grover et al, which was conducted in 2020 with a response rate of 24.8%, reported depression in 53% of the Indian ophthalmic surgeons.^[14]^ Depression was also discovered in 32.6% of all studied ophthalmologists practicing or training in India in 2020 and varied severities of mild, moderate, and severe depression were identified at 21.4%, 6.9%, and 4.3%, respectively.^[13]^ Additionally, 64.7% of all Turkish physicians who participated in 2020^[21]^ and 50.4% of Chinese physicians with the response rate of 68.7% in the study year of 2020^[22]^ had symptoms of depression during the COVID-19 pandemic. The higher percentage of depression in our study as compared with other reports could be attributed to the longer working hours of Iranian ophthalmologists as well as the high incidence and mortality rates of coronavirus disease in our country. It should also be considered that the various levels of severity of depression were all discovered in our study, as compared to the Indian Study where most of the subjects had only mild depression.^[13]^

### Depression and Gender

In our study, no statistically significant difference was observed in the prevalence of depression between different genders which was in line with the study by Chambers et al.^[25]^ However, higher incidence of depression was identified among female doctors as revealed in the annual report by the World Health Organization (WHO), the annual global prevalence, reported by Cyranowski et al Ford et al, and the study conducted on the Indian surgeon ophthalmologists and Saudi Arabian ophthalmologists.^[13,24,25,26,27]^ It was also discovered in a study investigated by Elbay et al that the incidence of depression was reported higher among female physicians in his sample.^[21]^

### Depression and Age

In this study, it was revealed that a higher percentage of older ophthalmologists suffered from depression. Our findings were in contrast with the study by Khanna et al on Indian ophthalmologists that reported the decreasing odds of 3% for depression with increasing age.^[13]^ This difference in comparison may be attributed to longer practice and training program hours conducted among the older ophthalmologists which may lead to increased prevalence of physical and psychological pressures.

### Depression and Marriage

Although in our study there was no difference between married and single ophthalmologists regarding depression, Elbay et al discovered that being married was associated with less incidence of depression and other psychological problems such as anxiety and stress.^[21]^ Studies on physicians and Indian ophthalmologists also showed that being single was considered an influential factor in increasing the depressive symptoms during the COVID-19 outbreak.^[13,21]^

### Depression and Duration of Practice

The mean duration of practice among our study participants was 17.26 ± 10.86 years, which was not significantly associated with depression. However, Elbay et al found that higher levels of depression and other mental disorders were observed among physicians who, as a result of having less working experience, executed excessive working hours in rotating shifts with minimal rest periods.^[21]^

### Depression and Last Degree

We found a lower percentage of depression among ophthalmologists in vitreoretinal fellowship as compared to the general ophthalmologists. This lower percentage might be attributed to the fact that as most of the retinal diseases need urgent management, patients with different types of retinal pathologies were referred to the vitreoretinal fellowship ophthalmologists during the COVID-19 pandemic as was done in the past. Another reason for lower percentages of depression among vitreoretinal fellowship participants may be as a subspecialty group they have adapted to operating under these challenging conditions while managing most of these complicated retinal diseases. On the contrary, Almater et al reported that depressive symptoms were significantly higher in fellows as compared to residents and consultants among the Saudi Arabian ophthalmologists.^[27]^ This discrepancy can be related to the different facilities which have been provided for different grades of ophthalmologists in different countries.

### Depression and Types of Services 

No statistically significant association was observed between depression and different services presented by the ophthalmologists. However, a higher level of depression was reported among in- training Indian ophthalmologists because of their concern regarding their training or profession challenges and getting their living. ${}^{13]}$ Almater et al did not find any difference between ophthalmologists who were working in the specialized eye centers and those who were practicing in general hospitals in terms of the depression incidence.^[27]^

### Depression and Concerns about Training/Profession and Income

The substantial record of depressed ophthalmologists is those that had serious concerns about their training/profession and income in this pandemic period, which proved to be an additional influence in maximizing depression. This finding was also reported in United Kingdom, Saudi Arabia, and India illustrating the significant impact of coronavirus disease on the ophthalmology-training program.^[13,16,28]^ The reduction of training courses for patient examination and surgeries at the educational hospitals due to the decreased number of referral patients and communication restrictions as a result of healthcare protocols contributed toward the negative impact on the ophthalmology training program. A higher level of depression was also reported among in-training Indian ophthalmologists because of their concern regarding their training or profession challenges and earning to sustain their living costs.^[13]^

### Association of Different factors with Depression

In assessing the prevalence of depression during the COVID-19 pandemic as it relates to multiple factors, it was determined that no inherent factors of coronavirus, communication problems, financial challenges, distress due to losing relatives or isolation were found to be influential. Higher percentages of depression was found even among patients with no prior history of psychological problems, so the direct impact of coronavirus on depression was able to be isolated. Our analysis shows that depression was discovered in ophthalmologists who were somewhat concerned as compared to those having serious concerns about their training/profession (OR = 0.024, 95%CI: 0.086–0.672; *P* = 0.007) while those with no concerns about their income had lower association with depression (OR: 0.065; 95% CI: 0.005–0.91; *P* = 0.042).

### Depression in Regions with Different levels of Risk of Coronavirus Disease

All ophthalmologists located in the low-risk regions had moderate depression and most of the participants (47.1%) located in the moderate-risk region had mild depression. However, we noticed higher percentages of severe depression in ophthalmologists located in the high-risk regions. It has been reported that ophthalmologists are considered as one of the frontline physicians with the high risk of contracting coronavirus disease, due to their close proximity toward patients during vision testing, slit lamp, and fundus examinations.^[27]^ Consequently, the application of personal protective equipment is necessary during patients' examination. In addition, supportive proceedings by the government, health administrations, and the Iranian Society of Ophthalmology should also be considered, particularly in the high-risk regions. The usage of the standard PHQ-9 questionnaire as our study tool can be a strength while the cross-sectional study design can be considered as our study limitation. Low response rate can also be taken into account as another limitation of the present study in spite of the sending several reminders, which can be attributed to the common taboo among our study population regarding the mental health problems.

In conclusion, high prevalence of depression was observed among older aged Iranian ophthalmologists living in high-risk contaminated regions who possessed serious concerns with respect to their training/profession and income. It is recommended that the health policymakers of Iran pay more attention to ophthalmologists who experience the aforementioned factors.

##  Availability of Data and Materials 

The datasets used and analyzed during this study can be made available from the corresponding authors upon reasonable request.

##  Financial Support and Sponsorship

There were no sources of funding for the research.

##  Conflicts of Interest

The authors report no conflict of interest.
